# Causality Evaluation of Drug-Induced Liver Injury in Newborns and Children in the Intensive Care Unit Using the Updated Roussel Uclaf Causality Assessment Method

**DOI:** 10.3389/fphar.2021.790108

**Published:** 2021-12-20

**Authors:** Ling Ye, Zeying Feng, Longjian Huang, Chengjun Guo, Xiong Wu, Li He, Wei Tan, Yi Wang, Xuehong Wu, Biwen Hu, Tong Li, Guoping Yang, Guo Chengxian, Qingnan He

**Affiliations:** ^1^ Center of Clinical Pharmacology, The Third Xiangya Hospital, Central South University, Changsha, China; ^2^ Youjiang Medical University for Nationalities, Baise, China; ^3^ School of Applied Mathematics, Guangdong University of Technology, Guangzhou, China; ^4^ Easier Data Technologies Co., Ltd, Changsha, China; ^5^ Department of Pediatrics, The Third Xiangya Hospital, Central South University, Changsha, China; ^6^ Department of Neonatology, Maternal and Child Health Hospital of Guangxi Zhuang Autonomous Region, Nanning, China; ^7^ School of Computer Science and Engineering, Central South University, Changsha, China; ^8^ Hunan Creator Information Technology Co., Ltd, Changsha, China

**Keywords:** drug-induced liver injury, newborns, children, critically ill, China, updated RUCAM, Roussel Uclaf causality assessment method

## Abstract

**Purpose:** Drug-induced liver injury (DILI) is a common adverse reaction in the clinic; however, there are relatively few reports of DILI in critically ill newborns and children. Making use of the Pediatric Intensive Care database (PIC), this study identifies which drugs are related to DILI in neonates and children in China.

**Methods:** Using the PIC, we screened for patients whose liver was suspected of being injured by drugs during hospitalization. The medicine they used was then assessed by the Roussel Uclaf Causality Assessment Method (RUCAM). At the same time, we also collated drug combinations that may affect CYP (Cytochrome P) enzyme metabolism, which may cause DILI.

**Results:** A total of 13,449 patients were assessed, of whom 77 newborns and 261 children were finally included. The main type of liver injury in neonates was mixed (83.1%), while the hepatic injury types of children were mostly distributed between hepatocellular (59.4%) and cholestatic (28.4%). In terms of the RUCAM assessment, the drugs that were most considered to cause or be associated with hepatic injury in newborns were medium and long chain fat emulsions (17%), sodium glycerophosphate (12%), and meropenem (9%); while omeprazole (11%), methylprednisolone sodium succinate (10%), and meropenem (8%) were the primary culprits of DILI in children. Drug combinations frequently seen in neonates that may affect CYP enzyme metabolism are omeprazole + budesonide (16.9%), dexamethasone + midazolam (10.4%), and midazolam + sildenafil (10.4%). In children, the commonly used drug combinations are fentanyl + midazolam (20.7%), ibuprofen + furosemide (18.4%), and diazepam + omeprazole (15.3%).

**Conclusions:** In this study, medium and long chain fat emulsions and sodium glycerophosphate have been strongly associated with DILI in newborns, while omeprazole and methylprednisolone sodium succinate play an important role in the DILI of children. Also, attention should be paid to the effect on CYP enzymes when using multiple drugs at the same time. In future DILI cases, it is advisable to use the latest RUCAM for prospective study design so that complete case data and high RUCAM scores can be collected.

## Introduction

Drug-induced liver injury (DILI) is defined as a liver injury caused by various medications, herbs, or other xenobiotics, leading to abnormalities in liver tests or liver dysfunction with the reasonable exclusion of other etiologies (Vuppalanchi et al., 2007). DILI is one of the most common adverse drug reactions, showing elevated serum transaminase and bilirubin when mild, but causing acute liver failure, and even death, if it is severe. DILI represents 3.5% of all inpatients due to jaundice (Björnsson, 2013) and accounts for 11% of the acute liver failure cases in America (Lucena, 2020). Regrettably, there is little clinical research data about DILI in newborns or children, and most comes from small-scale clinical observations in China. DILI is an under recognized cause of pediatric liver diseases. Pediatric DILI is relatively rare compared to DILI in adults and is infrequently reported (only 1% of total) as a suspected ADR (Adverse Drug Reaction) in children and adolescents (Ferrajolo, 2010).

The concomitant use of two or more drugs is very common in critically ill neonatal and pediatric patients. When two or more drugs are used together and act on the same enzyme, the metabolism of the drugs can be affected, resulting in accumulation of the medicine, which may cause liver damage. A combination of drugs acting on the same metabolic enzyme mainly affects the absorption, distribution, metabolism, and excretion of the drugs. Many metabolic pathways can be inhibited by co-administered drugs. According to current statistics, more than 90% of the metabolic drug interactions in clinical practice are caused by changes in CYP enzyme activity (Liu and Hong, 2009). Changes in the activity of hepatic drug enzymes at different stages in neonates and children can affect the metabolism of the drug, thus increasing or decreasing its toxicity. Without a doubt, CYP are the most significant enzymes of phase I metabolism. These enzymes metabolize 70–80% of drugs in the body, and these enzyme-mediated metabolisms are often the basis for drug interactions (Zanger and Schwab, 2013). Of the potentially hepatotoxic drugs, most are metabolized by CYP and a few by pathways involving non-CYP enzymes (Teschke and Danan, 2021). The most important forms of CYP include CYP1A1, CYP1A2, CYP2A6, CYP2B6, CYP2C8, CYP2C9, CYP2C19,CYP2D6, CYP2E1, CYP3A4, and, in newborns and children, CYP3A7 (Hines, 2008). Within these, the activities of CYP1A2, CYP2C9, CYP2C19, CYP2D6, CYP2E1, and CYP3A4 enzymes differ significantly in neonates, children, and adults, and play a pivotal role in drug metabolism. Thus, we collected information relating to the drugs associated with these six enzymes in patients.

The increase in the number of newborns and children is an unchangeable outcome of the gradual opening of the Family Planning Policy in China and the comprehensive opening of the Three-child Policy in particular. The incidence of DILI in children has an upward trend as awareness grows. However, there is a lack of comprehensive information about DILI in children and newborns in China. Therefore, this study aimed to access information about which drugs may be associated with hepatic injury in the newborn and pediatric population using the Pediatric Intensive Care database (PIC).

## Methods

### Data Source

This is a retrospective study. We selected cases from all patients in the PIC database (http://pic.nbscn.org/), which is a large pediatric-specific, single-center, bilingual database containing information relating to children admitted to the intensive care unit at a large children’s hospital in Zhejiang, China from 2010 to 2019. The data available in the PIC database includes demographic information, laboratory test results, observation program results during the patient’s hospitalization, vital signs, drug use records, and structured symptoms recorded while the patient was under supervision. All patients’ information, including demographics, laboratory test results, symptoms, and medications, were collected from the PIC database.

### Data Extraction

We defined newborns as babies aged 1–31 days old. The age range of children was from 32 days to 14 years old. The criteria for liver injury was: 1) alanine aminotransferase (ALT) ≥ times; 2) alkaline phosphatase (ALP) ≥2 times the upper limit of normal value (ULN) (Danan and Teschke, 2015). We then excluded patients with known primary liver diseases, such as autoimmune hepatitis, or other diseases that could confound the diagnosis. Hepatic injury was categorized as hepatocellular, cholestatic, and mixed, based on the value of R (Danan and Benichou, 1993; Danan and Teschke, 2015). The R value is equal to the ratio of the serum ALT and its maximum value to the ALP and its maximum value. If the R value was ≥5, we considered it to be the hepatocellular type; if the R value was ≤2, we defined the type as cholestatic; if the R value was between 2 and 5, then the type was classified as mixed. After collecting information on all the medicines used by the included patients, we used the Roussel Uclaf Causality Assessment Method (RUCAM) (Danan and Teschke, 2015) to evaluate the relationship between the drugs used during the period of hospitalization and the patient’s liver injury. RUCAM is the most widely used scale around the world (Danan and Benichou, 1993; Danan and Teschke, 2015), and the RUCAM values were categorized as “highly probable” (≥9), “probable” (6–8), “possible” (3–5), “unlikely” (1–2), or “excluded” (≤0). All evaluations were performed independently by the three authors. The website Hepatoxic (http://www.hepatox.org/) and drug instructions were used as the drug information source to verify the relationship between the drugs and hepatotoxicity.

### Combination of CYP Metabolizing Enzyme-Related Drugs

After searching the literature for CYP-related drugs, we compared and summarized the following CYP enzymes, which differ in their activities in neonates, children and adults: CYP1A2, CYP2C9, CYP2C19, CYP2D6, CYP2E1, and CYP3A4. When drugs related to these metabolizing enzymes are combined, drug accumulation due to drug-drug interactions may also cause liver injury. We extracted all relevant drug combinations from all patients’ medication information.

### Effect of DILI on Length of Stay and Prognosis

We selected several diseases that occur most frequently in neonates and children with DILI. The length of stay and mortality of patients with DILI under these diseases were then collected and counted, and these were compared with patients who had the same diseases but without DILI in the PIC.

### Statistical Analysis

Values are presented as percentages, medians, or means ± standard deviations. Data with normal distribution were subjected to an independent sample *t*-test, while data with non-normal distribution were subjected to the Wilcoxon rank sum test for comparison of two independent samples and a chi-square test or Fisher’s exact test for categorical variables. All of the calculations were performed by SPSS 17.0. *p* < 0.05 was considered statistically significant.

## Results

### The Baseline Characteristics of DILI

**FIGURE 1:**
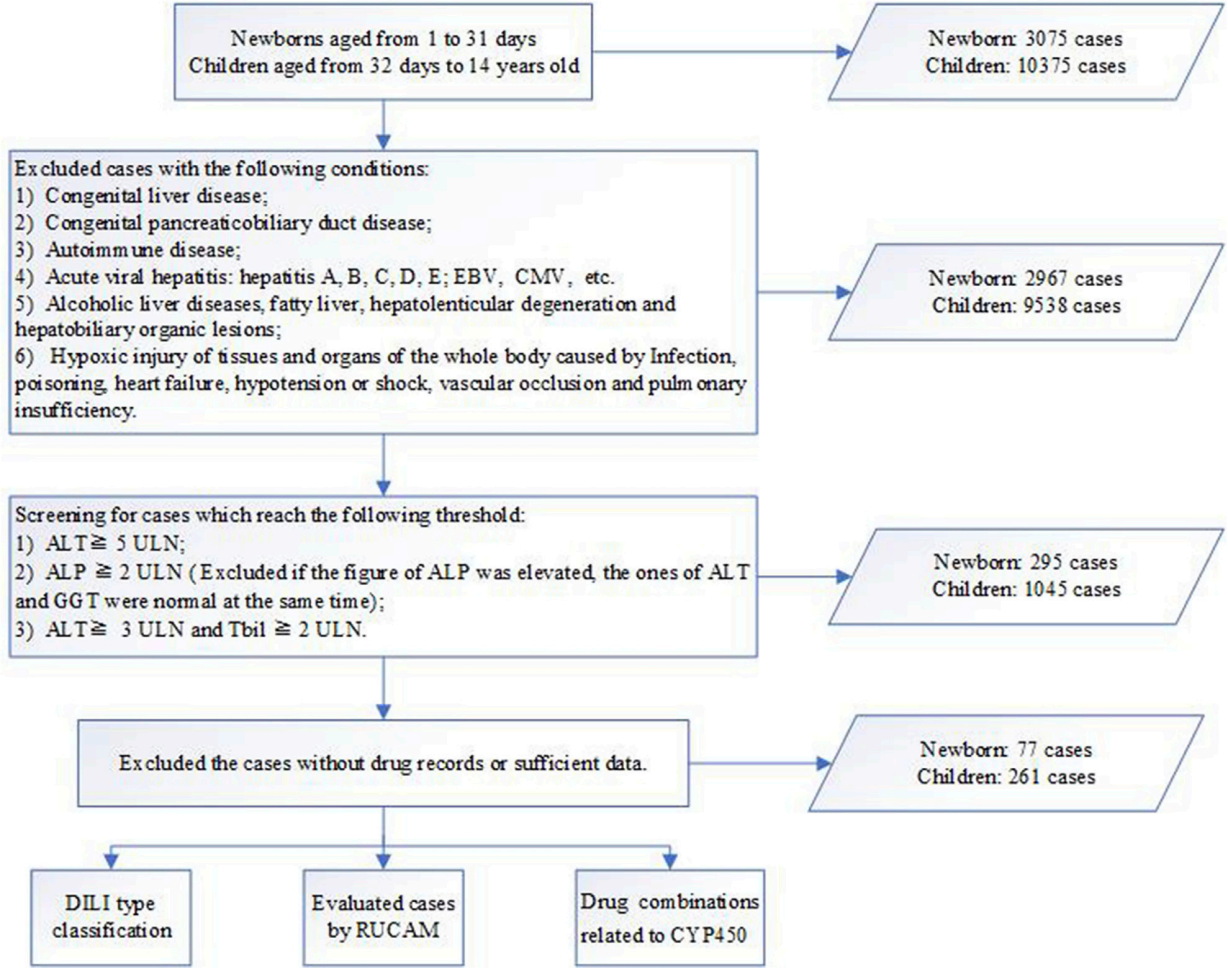
Workflow for diagnosis, classification and evaluation of drug-induced liver injury (DILI). ALT, alanine transaminase; ALP, alkaline phosphatase; Tbil, total bilirubin; ULN, upper limit of normal; RUCAM, updated Roussel Uclaf Causality Assessment Method; DDIs, drug-drug induced interactions.

A total of 13,449 patients were assessed during the study, of which 3,075 were newborns and the remaining were children. In this study, the frequency of DILI was 2.5% in newborns and 2.5% in children. The flow chart of the case ascertainment of DILI patients is shown in Figure 1.

Table 1 shows the characteristics of the study population. In newborns (*n* = 77), the majority of patients were male, with an average age of 8.5 days. The main initial diagnoses were respiratory diseases (22.1%) and digestive diseases (20.8%). The average length of hospitalization was 56 days. There were 2 (2.6%) patients whose clinical outcome was death. In children (*n* = 261), the proportion of male patients was about half, with an average age of 2.83 years. The main admission diagnoses for children were cardiovascular diseases (24.5%) and respiratory diseases (22.6%), 31 (11.9%) of whose clinical outcome was death. The average number of days of hospitalization in children was 30 days.

**TABLE 1:** Baseline characteristics of included patients.

	Newborns (*n* = 77)	Children (*n* = 261)
Age (days/years)	8.5 ± 9.8	2.83 ± 3.7
Male (gender)	47 (61.0%)	150 (57.5%)
Initial diagnosis		
Cardiovascular disease	6 (7.8%)	64 (24.5%)
Respiratory disease	17 (22.1%)	59 (22.6%)
Digestive disease	16 (20.8%)	17 (0.07%)
Ohers	38 (49.4)	121 (46.4%)
Hospitalization days	56 ± 40.4	30 ± 25.7
Clinical outcome		
Death	2 (2.6%)	31 (11.9%)

### Liver Test Abnormalities and DILI Phenotypes

Based on the R value, we divided the patients into three types. Among newborns, 9.1% were considered hepatocellular, 7.8% were cholestatic, and 83.1% were mixed. Most children were considered hepatocellular (59.4%), with 28.4% cholestatic and only 12.2% mixed. The distribution of liver injury types in newborns and children was distinct (*p* < 0.01). We compared the classification results with other similar studies (Supplementary Table S1), and found that there was some difference.

### Implicated Drugs and Causality Assessment

We used the updated RUCAM to assess the relationship between the patient’s medication and the DILI he/she was suffering from (Danan and Teschke, 2015), as well as the Hepatoxic website (http://www.hepatox.org/) and drug instructions to confirm and supplement the information about the medication. The likelihood outcomes of the updated RUCAM are presented in Table 2. Among all neonatal cases, 41 cases (53.2%) of DILI causative agents were classified as “probable”, and 34 cases (44.2%) were “possible”. Among all children’s cases, 167 cases (64%) of DILI causative agents were classified as “highly probable”, and 86 cases (33%) were “possible”.

**TABLE 2:** The characteristics of DILI.

	Newborns (*n* = 77)	Children (*n* = 261)	*p-*value
Type (based on R^a^ ratio)			
Hepatocellular	7 (9.1)	155 (59.4)
Cholestatic	6 (7.8)	74 (28.4)
Mixed	64 (83.1)	32 (12.2)
			<0.001
Peak level of laboratory findings			
ALT (U/L)	910 (226–2,051)	624 (201–10,266)	0.412
ALP(U/L)	411 (252–2,045)	361 (250–1,888)	0.372
RUCAM score, mean (median)	2.70 (3)	2.54 (2)	<0.001
Highly probable	2 (2.6)	8 (3.0)	
Probable	41 (53.2)	167 (64.0)	
Possible	34 (44.2)	86 (33.0)	
			0.202
Number of drugs used during hospitalization	23 ± 14.0	26 ± 14.0	0.124

a R= (ALT value/ALT ULN)/(ALP value/ALP ULN). R > 5 = hepatocellular, R < 2 = cholestatic, R between 2 and 5 = mixed.

Values are presented as number (%) or median (range) or Mean ± SD.

**FIGURE 2:**
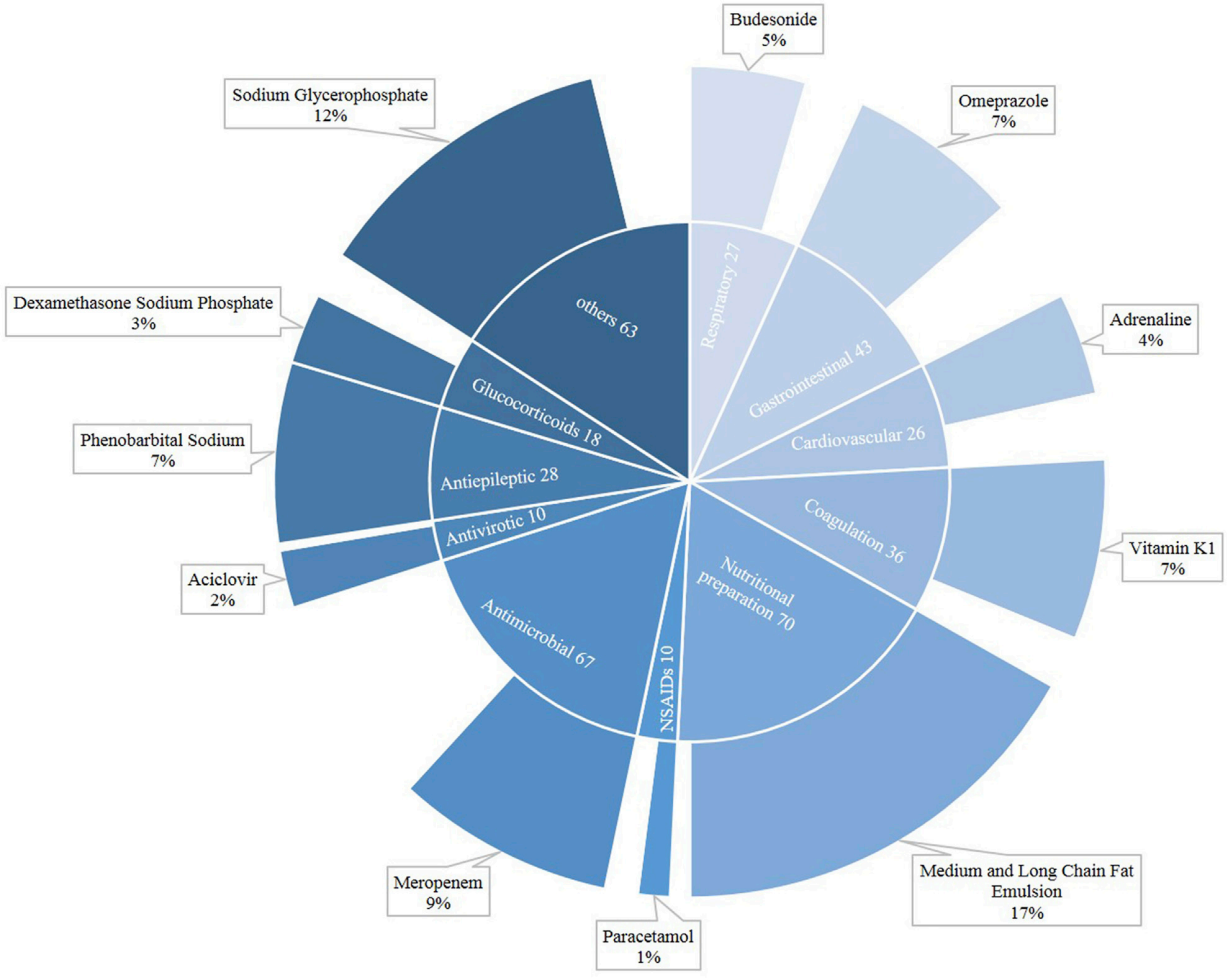
Drug categories for neonatal drug-induced liver injury.

Figure 2 shows the distribution of drugs that caused neonatal DILI. In neonates, a total of 398 drugs were suspected of being responsible for DILI. In newborns, the major categories were nutritional preparation (*n* = 70), followed by antimicrobial (*n* = 67) and gastrointestinal (*n* = 43). Among the nutritional preparations, the most common agent was the medium and long chain fat emulsion (17%), while meropenem (9%) was the main antimicrobial, and sodium glycerophosphate also played an important role (12%).

**FIGURE 3:**
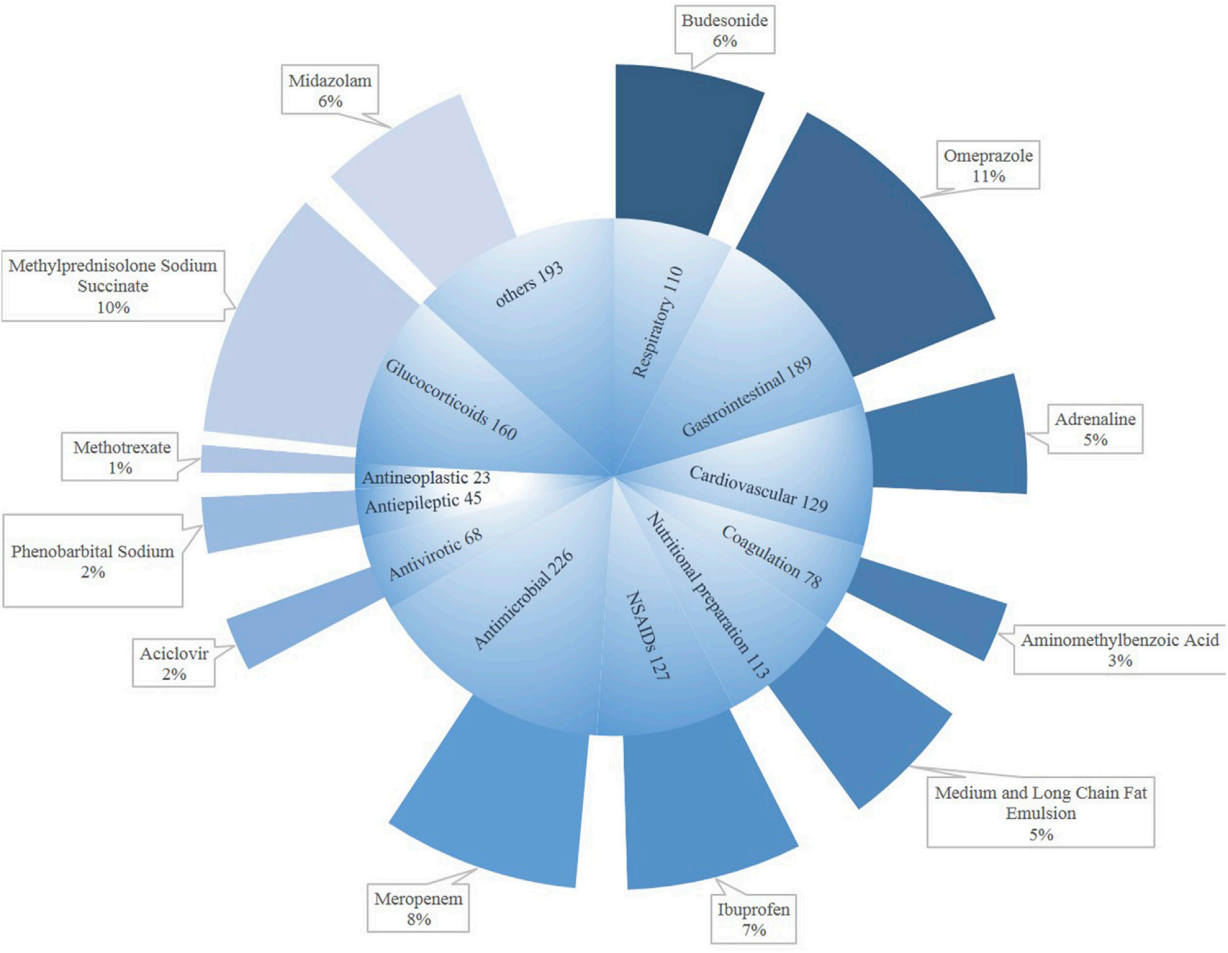
Drug categories for DILI in children.

Figure 3 shows the drug categories for DILI in children. The numbers of drugs regarded as causative agents was 1,461. Among them, major classes were antimicrobial (*n* = 226), followed by gastrointestinal medication (*n* = 189) and glucocorticoids (*n* = 160). The bulk antimicrobial drug was meropenem (8%), as for the newborns. The main causative gastrointestinal medication was omeprazole (11%), with methylprednisolone sodium succinate being a large component (10%). Meanwhile, we could not ignore ibuprofen (7%).

In other related studies (Supplementary Table S2), antibacterial drugs, NSAIDs, Chinese herbal medicine, and nutritional preparations were the main implicated classes of agents that cause hepatic injury.

### Drug Combinations Related to CYP450

The drug combinations frequently seen in neonates are omeprazole + budesonide (16.9%), dexamethasone + midazolam (10.4%), and midazolam + sildenafil (10.4%) (Table 3). In children, the commonly used drug combinations are fentanyl + midazolam (20.7%), ibuprofen + furosemide (18.4%), diazepam + omeprazole (15.3%), omeprazole + budesonide (12.3%), omeprazole + methylprednisolone (9.6%), budesonide + methylprednisolone (8.8%), and fentanyl + methylprednisolone + midazolam (8.8%). It is clear that the combination of drugs in children with severe illness is more numerous and complex.

**TABLE 3:** Drug combinations related to CYP.

	Newborns	Children
CYP1A2	Omeprazole + Phenobarbital Sodium (4); Erythromycin + Phenobarbital Sodium (2); Omeprazole + Erythromycin (2); Omeprazole + Caffeine (1) Omeprazole + Lidocaine (1); Lidocaine + Erythromycin (1).	Omeprazole + Phenobarbital Sodium (4); Erythromycin + Phenobarbital Sodium (2); Omeprazole + Erythromycin (2); Omeprazole + Caffeine (1); Lidocaine + Erythromycin (1).
CYP2C9	Phenobarbital Sodium + Ibuprofen (1); Phenobarbital Sodium + Paracetamol (1); Paracetamol + Ibuprofen (1).	Ibuprofen + Furosemide (48); Paracetamol + Furosemide (26); Paracetamol + Furosemide (15); Phenobarbital Sodium + Furosemide (13); Paracetamol + Ibuprofen + Furosemide (5); Phenobarbital Sodium + Ibuprofen (3); Phenobarbital Sodium + Ibuprofen + Furosemide (2); Indometacin + Furosemide (1); Furosemide + Warfarin (1); Indometacin + Furosemide + Ibuprofen (1).
CYP2C19	Omeprazole + Sodium Valproate (1)	Diazepam + Omeprazole (40); Sodium Valproate + Diazepam (9); Voriconazole + Omeprazole (7); Omeprazole + Sodium Valproate (7); Diazepam + Voriconazole (6); Omeprazole + Voriconazole (4); Sodium Valproate + Diazepam + Omeprazole (3); Sodium Valproate + Voriconazole (1).
CYP3A4	Omeprazole + Budesonide (13); Dexamethasone + Midazolam (8); Midazolam + Sildenafil (8); Omeprazole + Midazolam (7); Omeprazole + Dexamethasone + Fentanyl + Midazolam (5); Budesonide + Dexamethasone (5); Budesonide + Midazolam (5); Fentanyl + Midazolam (4); Fluconazole + Caffeine (4); Spironolactone + Midazolam + Sildenafil (4); Omeprazole + Dexamethasone + Midazolam (2); Omeprazole + Fentanyl + Midazolam (2); Budesonide + Spironolactone + Sildenafil (2); Diazepam + Midazolam (2); Erythromycin + Midazolam (2); Methylprednisolone + Midazolam (2); Lidocaine + Midazolam (2); Spironolactone + Midazolam (2); Azithromycin + Caffeine (1).	Fentanyl + Midazolam (54); Omeprazole + Budesonide (32); Omeprazole + Methylprednisolone (25); Budesonide + Methylprednisolone (23); Fentanyl + Methylprednisolone + Midazolam (23); Omeprazole + Fentanyl + Midazolam (12); Budesonide + Salbutamol (11); Diazepam + Midazolam (11); Omeprazole + Dexamethasone (10); Omeprazole + Diazepam (10); Omeprazole + Midazolam (10); Budesonide + Midazolam (10); Sodium Valproate + Midazolam (9); Budesonide + Fentanyl + Midazolam (9); Dexamethasone + Voriconazole (9);Paracetamol + Methylprednisolone (9); Diazepam + Fentanyl + Midazolam (8); Omeprazole + Dexamethasone + Fentanyl + Midazolam (7); Budesonide + Spironolactone (7); Ondansetron + Vindesine + Dexamethasone (6); Sodium Valproate + Diazepam (6); Diazepam + Methylprednisolone (6); Vindesine + Dexamethasone (6); Omeprazole + Diazepam + Fentanyl + Midazolam (5); Omeprazole + Fentanyl + Methylprednisolone + Midazolam (5); Omeprazole + Methylprednisolone + Midazolam (5); Budesonide + nifedipine (5);Digoxin + Spironolactone (5); Dexamethasone + Midazolam (5); Lidocaine + Midazolam (5).

### The Impact of DILI on Length of Stay and Prognosis

From the data in Table 4, we can see that DILI significantly prolongs the length of stay in the three conditions of neonatal respiratory distress syndrome (*p* = 0.039), neonatal asphyxia (*p* = 0.010), and gastrointestinal dysplasia (*p* = 0.002), and the difference with the non-DILI group is statistically significant. There was no statistical difference in length of stay between the DILI and non-DILI groups for the three diseases of pneumonia (*p* = 0.367), enterocolitis (*p* = 0.085), and congenital heart disease (*p* = 0.128), but the median length of stay was seen to be greater in the DILI group than in the non-DILI group for all three of these diseases. In six diseases including neonatal respiratory distress syndrome, there was no apparent difference between the DILI and non-DILI groups.

**TABLE 4:** Comparison of days of hospitalization and mortality between the neonatal DILI and non-DILI groups.

	Neonatal respiratory distress syndrome		P	Neonatal asphyxia		P	Neonatal pneumonia		P	Dysplasia of digestive system		P	Enteritis		P	Congenital heart disease		P
	DILI group	Non-DILI group		DILI group	Non-DILI group		DILI group	Non-DILI group		DILI group	Non-DILI group		DILI group	Non-DILI group		DILI group	Non-DILI group	
Days of hospitalization	41 (17–44)	15 (0–71)	0.039	27 (14–79)	11 (0–77)	0.010	32 (9–63)	19 (0–110)	0.367	65 (21–222)	22 (1–88)	0.002	63 (28–94)	26 (0–105)	0.085	28 (16–57)	20 (0–92)	0.128
Mortality	0	4%	1.000	0	15.2%	1.000	25%	6.2%	0.247	0	9.7%	1.000	0	13.6%	1.000	0	20.6%	0.581

In Table 5, there is a significant and statistically meaningful difference in the length of stay between the DILI and non-DILI groups in children with pneumonia (*p* = 0.039) and congenital heart disease (*p* = 0.010). In terms of mortality, the differences between the DILI and non-DILI groups were not statistically significant for any of the four diseases.

**TABLE 5:** Comparison of days of hospitalization and mortality between DILI and non-DILI groups in children.

	Pneumonia		P	Dysplasia of digestive system		P	Enteritis		P	Congenital heart disease		P
	DILI group	Non-DILI group		DILI group	Non-DILI group		DILI group	Non-DILI group		DILI group	Non-DILI group	
Days of hospitalization	28 (2–74)	13 (0–335)	<0.001	20 (7–64)	15 (4–175)	0.205	16 (6–150)	14 (0–135)	0.210	26 (3–58)	13 (0–146)	<0.001
Mortality	20%	14.0%	0.274	0	0	1.000	16.7%	8.3%	0.320	9.1%	3.1%	0.149

## Discussion

In this study, we screened the children with DILI in the past 10 years using the PIC database, collecting their medication information and evaluating the drugs by RUCAM. In this retrospective, single center study, antibiotics and nutritional preparation agents were considered to be causative drugs of DILI in newborns. Antibiotics and gastrointestinal medicine were found to give rise to DILI in children during hospitalization. Meropenem was the most common individual antibiotic drug. There was not much difference in the peak level of laboratory findings and the number of drugs used during hospitalization between newborns and children with DILI. Neonates and children varied in the type of DILI. After dividing the patients by the R ratio, we found that DILI in newborns was dominant in the mixed type, while children were dominant in the hepatocellular type. The distribution of types of liver injury in critically ill children in our study differed slightly from the types summarized in other studies. After collecting information on the drugs associated with important CYP enzymes, we compiled a summary of the combinations that are commonly seen in clinical practice. The combination of these drugs may lead to competition for the same enzyme’s site of effect or a conflict of action, which may in turn result in the accumulation of the drug and cause liver injury. Finally, we compared the impact of length of stay and prognosis of patients with DILI in some common diseases.

The data available in the PIC database includes laboratory measurements, charted observation during patient’s hospitalization, structured symptoms extracted from notes, and vital signs recorded while the patient was in the operating room, thus providing reliable and abundant data for our research. DILI patients in the PIC require investigator-directed screening and assessment using RUCAM. Since this was not a prospective study, some of the data were missing and also made the overall score of our DILI patients low.

For the first time, we focused on the issue of drug-related hepatic injury in neonates, as opposed to children and adults, who are often studied, and also compared the results to children. The neonate is a very special patient population and a unique recipient (Morselli, 1976), as a result of immaturity at birth and the daily evolution of many metabolic functions (De Gregori, 2009). As a consequence of the incomplete maturity of such vital functions at birth, neonates show significant differences in absorption, distribution, metabolism, and excretion, compared to adults (Anderson, 2002). The main types of liver injury in neonates are mixed and cholestatic, which may also be related to the way we currently determine the type of liver injury, using the R value. Unlike the critically ill neonates in this study, the distribution of liver injury types in other studies was predominantly hepatocellular. That is to say, the major types of hepatic injury in severely ill newborns in China differ from those in Asian children and adults, as well as from those in American children and adults. Except for the distribution of adult liver injury types in the United States, which had similar proportions of cholestatic and mixed types, the number of patients with mixed types was greater than those with cholestatic types in all other studies. This is also dissimilar to the distribution of liver injury types in children with severe disease in our study in which the number of cholestatic types was greater than the number of mixed types. The laboratory biochemical assessments for DILI including ALP are not quite appropriate in newborns. ALP is expressed in liver tissue and is increased in hepatic dysfunction, but increased serum ALP can also be a result of bone growth and excessive enzyme secretion by osteoblasts (Magnusson et al., 1995), which may cause some inaccuracy in our results. The predominant type of liver injury in children was roughly the same as in other studies, with the vast majority being hepatocellular.

In other studies in China (Shen, 2019; Zhang, 2019), the drugs that mainly cause liver damage are Chinese herbs, drugs for tuberculosis, antibiotics, and NSAIDs, and antibiotics are responsible for most liver damage in studies of other countries (Chalasani, 2015; DiPaola, 2019; Kang, 2020). In our newborns, the main causes of liver damage were nutritional agents and antibiotics; in our pediatric patients, the main cause was antimicrobials and digestives system drugs. This may be related to the common diseases in critically ill patients. In our study, gastrointestinal and respiratory diseases were common in patients, who required dietary supplements and more advanced antibiotics; the demand for traditional Chinese herbal medicine would also be reduced. Moreover, newborns and children are at an essential period of growth and development, and the diseases of critically ill children often lead to the malabsorption of nutrients that affect their development, thus creating a greater need for nutritional preparations. This result suggests that pediatricians and pharmacists should also pay more attention to the liver function indicators of critically ill newborns and children when using nutritional preparations. At the same time, we compared the results with the latest published papers on the subject (Teschke, 2018). The top five DILI-causing drugs in this latest report were amoxicillin clavulanic acid, flucloxacillin, atorvastatin, disulfiram, and diclofenac, which differed significantly from our findings. This may be related to the large differences in the study populations in the two studies.

As seen in Tables 4, 5, the length of hospital stay was remarkably or slightly longer in the DILI groups than in the non-DILI groups. The treatment of patients with liver protection, while not interfering with the treatment of other diseases, or DILI aggravated pre-existing conditions, which made the treatment of critically ill patients more complicated and prolonged the treatment time. However, we found that DILI did not dramatically increase the mortality rate of neonates and children with the same disease. This may be due to the following reasons: 1. Statistical bias caused by the small number of patients in the DILI groups; 2. Detection and measures taken by health care professionals to effectively stop the deterioration of the diseases; 3. The mild degree of DILI in patients, which didn’t cause irreversible and severe damage to liver function.

It is noteworthy that chronic liver injury did not develop in any of our patients, and the mortality was lower than 1% in both neonates and children. The cases of death all related to other etiologies, such as organ dysfunction or tumor, which means that hepatitis and liver injury did not have much to do with the death in these cases. Most patients with DILI were expected to recover or improve their condition after withdrawing the suspected drug and beginning supportive treatment.

Hines et al. suggested that there are three patterns of the developmental expression of CYP: 1. Expression in the fetal liver, decreasing gradually with gestational age (e.g., the subtype CYP3A7); 2. Expression begins in the early neonatal period (e.g., CYP2D6 and CYP2E1); 3. Expression starts in late neonate development (e.g., CYP1A2 and CYP3A4) (Hines, 2013). The most abundant enzyme in the human liver is CYP3A4, which is widely considered to be involved in the metabolism of more than half of medicines (Wrighton and Stevens, 1992). CYP3A4 activities in the liver of neonates are much lower than in adults, resulting in lower metabolism and reduced clearance of antibiotics, antivirals, hormones, and other drugs in the liver, and the easy accumulation of drugs (Zhang, 2019). After birth, CYP1A2 activity towards its substrates, caffeine and theophylline, is low but reaches adult levels at 4–5 months (Kraus, 1993). One of the substrates of CYP2E1 is acetaminophen and, if glutathione is depleted, the enzyme irreversibly damages the liver tissue (Coen, 2015). The amount of this enzyme increases rapidly after roughly 3 months (Matalová et al., 2016). Co-administration of the same substrates of CYP2E1 was not found in this study. Neonatal CYP2D6 activity is only about 3–5% of that of adults, resulting in less hepatotoxic metabolites being converted when substrates are ingested and a lower incidence of DILI than in adults (Faa, 2012). Typical substrates for CYP2C9 and CYP2C19 include NSAIDs, sartans, proton pump inhibitor, warfarin, and propranolol. At around 5 months of age, about half of children reach adult levels (Matalová et al., 2016). That is to say, most enzyme activities in newborns and some children are lower than in adults. From the results of Table 3, we also found many drugs related to DILI that were summarized in this study, such as omeprazole and budesonide, which are frequently seen in neonatal species, and midazolam and ibuprofen which are commonly seen in children. According to our statistics, newborns and children both used at least five or more medications during their hospitalization. The concomitant use of drugs acting on the same enzyme can have some impact on drug metabolism and excretion, and may also be implicated in liver damage.

Our study has several limitations. First, it is a retrospective and single center study, which means a smaller sample size. Second, due to the lack of thresholds of hepatic injury in newborns and children, we have referred to the DILI standard for adults, which may lead to deviation in the screening results. Throughout the study, most of the research focused on DILI in patients during hospitalization (Ocete-Hita, 2017; Kang, 2020); thus, our research on outpatients is deficient.

Due to the particularity of neonates and children, they are seldom of concern in DILI. However, DILI is a potential but preventable cause of hepatic injury, and drug interactions in multiple drugs combination are also a problem that cannot be ignored. More accurate standards of diagnosis and higher attention are urgently needed in DILI in neonatal or children.

## Conclusion

We used the updated RUCAM to assess causality and found medium and long chain fat emulsions, sodium glycerophosphate and meropenem to be the culprits of DILI in newborns. When using omeprazole, methylprednisolone sodium succinate, and meropenem in children, physicians and pharmacists must also be careful about the potential for liver injury. If multiple drugs are used together, attention must be paid to the effects on CYP enzymes.

## Data Availability

The original contributions presented in the study are included in the article/Supplementary Materials, further inquiries can be directed to the corresponding authors.
