# Supplementary material for: Causality Evaluation of Drug-Induced Liver Injury in Newborns and Children in the Intensive Care Unit Using the Updated Roussel Uclaf Causality Assessment Method
Source: Front Pharmacol. 2021 Dec 20;12:790108. doi: 10.3389/fphar.2021.790108 (PMC8721278; doi:10.3389/fphar.2021.790108)
Supplement: Supplementary file 1 [file DataSheet1.docx]

|  | A study in mainland China^[1]^  (n=25,927) | A hospital in China^[2]^  (n=187) | A hospital in KOREA^[3]^  (n=735) | A study in America^[4]^  (n=899) | A study in American children^[5]^  (n=57) |
| --- | --- | --- | --- | --- | --- |
| Age | All ages | 7(0-14) | 48.4±24.1 | 49.0±17.0 | 14.3(1.7-17.9) |
| Male | 50.8% | 62.0% | 35.6% | 41% | 33.3% |
| Type of injury |  |  |  |  |  |
| Hepatitis | 51.4% | 77.0% | 86.3% | 53.8% | 82.0% |
| Cholestatic | 20.3% | 10.0% | 1.6% | 23.4% | 8% |
| Mixed | 28.3% | 13.0% | 12.1% | 22.8% | 10% |

[1] Shen T, Liu Y, Shang J, Xie Q, Li J, Yan M, et al. Incidence and Etiology of Drug-Induced Liver Injury in Mainland China. Gastroenterology. 2019;156:2230-41 e11.

[2] ZHANG Y, GUO Y, M. N, al． e. Drug － induced liver injury in children: An analysis of medication and clinical features. Journal of Clinical Hepatology. 2019;35:579-84.

[3] Kang Y, Kim SH, Park SY, Park BY, Lee JH, An J, et al. Evaluation of Drug-Induced Liver Injury Developed During Hospitalization Using Electronic Health Record (EHR)-Based Algorithm. Allergy Asthma Immunol Res. 2020;12:430-42.

[4] Chalasani N, Bonkovsky HL, Fontana R, Lee W, Stolz A, Talwalkar J, et al. Features and Outcomes of 899 Patients With Drug-Induced Liver Injury: The DILIN Prospective Study. Gastroenterology. 2015;148.

[5] DiPaola F, Molleston JP, Gu J, Cirulli ET, Chalasani N, Barnhart H, et al. Antimicrobials and Antiepileptics Are the Leading Causes of Idiosyncratic Drug-induced Liver Injury in American Children. Journal of pediatric gastroenterology and nutrition. 2019;69:152-9.

Supplementary Table 1 The Type of Injury in Other Studies

|  | A study in mainland China^[1]^  (n=25,927) | A hospital in China^[2]^  (n=187) | A hospital in KOREA^[3]^  (n=735) | A study in America^[4]^  (n=899) | A study in American children^[5]^  (n=57) |
| --- | --- | --- | --- | --- | --- |
| Study design | retrospective | retrospective | retrospective | prospective | prospective |
| Duration(years) | 2012-2014 | 2008-2017 | 2015-2016 | 2004 | 2004 |
| Top implicated drugs | TCM^[[1]](#endnote-1)^ or HDS^[[2]](#endnote-2)^ (26.81%), tuberculostatics (21.99%), antineoplastic or immunomodulators (8.34%) and antiinfectious (6.08%) | antibiotics(42%),  NSAIDs^[[3]](#endnote-3)^(56%), | antibiotics(43%),  chemotherapeutics  (19.2%),  acetaminophen  29 (6.9%),  TPN^[[4]](#endnote-4)^(5.3%) | antimicrobials(45%),  herbal and dietary supplements(16.1%),  cardiovascular agents(9.9%), central nervous system agents(9.1%)  anti-neoplastic agents(5.5%) | antimicrobial (50.9%), antiepileptic  (21.1%),  antineoplastic  (8.8%), psychotropic (8.8%) |

Supplementary Table 2 The Most Implicated Classess of Agents

1. traditional Chinese medicine [↑](#endnote-ref-1)
2. herbal and dietary supplements [↑](#endnote-ref-2)
3. nonsteroidal antiinflammatory drugs [↑](#endnote-ref-3)
4. total parenteral nutrition [↑](#endnote-ref-4)
